# Vaccination of Calves with the *Mycobacterium bovis* BCG Strain Induces Protection against Bovine Tuberculosis in Dairy Herds under a Natural Transmission Setting

**DOI:** 10.3390/ani12091083

**Published:** 2022-04-22

**Authors:** Pedro Ábalos, Nicolás Valdivieso, Bernat Pérez de Val, Martin Vordermeier, María Belén Benavides, Raúl Alegría-Morán, Karina Saadi, Mathias Wistuba, Camila Ortega, Nicole Sánchez, Patricio Retamal

**Affiliations:** 1 Facultad de Ciencias Veterinarias y Pecuarias, Universidad de Chile, Santiago 8820808, Chile; pabalos@uchile.cl (P.Á.); mbbv@veterinaria.uchile.cl (M.B.B.); ralegria@veterinaria.uchile.cl (R.A.-M.); mathias.wistuba@ug.uchile.cl (M.W.); camila.ortega@veterinaria.uchile.cl (C.O.); nicole.sanchez.m@ug.uchile.cl (N.S.); 2 Servicio Agrícola y Ganadero, Santiago 8330246, Chile; nicolas.valdivieso@sag.gob.cl (N.V.); karina.saadi@sag.gob.cl (K.S.); 3 IRTA, Programa de Sanitat Animal, Centre de Recerca en Sanitat Animal (CReSA), Campus de la Universitat Autònoma de Barcelona (UAB), Bellaterra, 08193 Barcelona, Spain; bernat.perez@irta.cat; 4 Department of Bacteriology, Animal and Plant Health Agency, Addlestone KT15 3NB, UK; martin.vordermeier@apha.gov.uk; 5 Centre of Excellence for Bovine Tuberculosis, Aberystwyth University, Aberystwyth SY23 3EE, UK

**Keywords:** BCG, calves, protection, tuberculosis, field trial

## Abstract

**Simple Summary:**

Bovine tuberculosis (bTB) is a zoonotic disease caused mainly by *Mycobacterium bovis*, of which control is based on culling infected animals and, without official compensations, is associated with major economic losses for milk and meat producers. The vaccination of cattle with the *M. bovis* Bacillus Calmette-Guerin (BCG) strain, as a strategy for bTB control, might attenuate this situation. The objective of this trial was to assess the efficacy of the BCG Russia strain in a cohort study performed under field conditions, with the vaccination of 501 calves in seven dairy farms, including 441 non-vaccinated control animals. Peripheral blood was collected at 6, 12 and 18 months post-vaccination, and infection status was determined using a diagnostic procedure which discriminates the infected amongst vaccinated animals. On average, the BCG vaccine showed a low but significant level of protection (22.4%) at the end of the trial, although diverse levels of protection and duration of immunity were observed between trial herds, suggesting that the efficacy of the BCG vaccination could be influenced by the general health condition of calves and their exposition to non-tuberculous mycobacteria. These results support the use of BCG as a complementary tool in the control of the disease in high prevalence areas.

**Abstract:**

Bovine tuberculosis (bTB) is a zoonotic disease caused mainly by *Mycobacterium bovis*, which is associated with major economic losses for milk and meat producers. The objective of this trial was to assess the efficacy of the BCG Russia strain in a cohort study performed under field conditions, with the vaccination of calves in seven dairy farms from a high prevalence area in central Chile. The trial was performed with 501 animals, subcutaneously vaccinated with 2–8 × 10^5^ colony-forming units of BCG, whilst 441 matched control animals received a saline placebo. Peripheral blood was collected at 6, 12 and 18 months post-vaccination, and infection status was determined using the IFNγ release assay in conjunction with the DIVA (Detecting Infected amongst Vaccinated Animals) antigens ESAT-6, CFP-10 and Rv3615c. The BCG vaccine showed a low but significant level of protection of 22.4% (95% CI 4.0 to 36.4) at the end of the trial. However, diverse levels of protection and a variable duration of immunity were observed between trial herds. This diverse outcome could be influenced by the general health condition of calves and their exposition to non-tuberculous mycobacteria. These results suggest that BCG vaccination of dairy calves in a natural transmission setting confers variable protection to animals against bTB in a high prevalence area.

## 1. Introduction

Tuberculosis is an infectious disease of chronic course that is related to multiple animal species, including humans. In animals, the main cause of tuberculosis is *Mycobacterium bovis*, a wide-host-range pathogen associated with zoonotic transmission, which can persist for a long time in the environment, especially in humid conditions, moderate temperatures, with sunlight protection, and in the presence of organic matter. Zoonotic transmission mainly occurs in occupational contexts or by raw milk consumption [1,2].

Bovine tuberculosis (bTB) is a worldwide-distributed disease, although the incidence may vary according to geographical, environmental, management and age-related factors [3]. The disease represents a threat to public health and significant economic losses due to lower productivity of animals, their constant replacement, veterinary assistance and carcass condemnations in slaughterhouses [4]. This specially affects the economy of low- to middle-income countries [5], where the disease is endemic, and the livestock industry and farmers do not receive any compensation for culling reactor animals, meaning that the test-and-slaughter strategy implemented in control programs in high prevalence areas is generally not affordable [6].

In dairy herds, cattle usually become infected during the first months of life, when calves are exposed to contaminated colostrum, milk or respiratory droplets from infected cows [3].

Human tuberculosis is within the leading causes of death and morbidity globally [7]. However, it is to some degree a preventable disease mainly through vaccination with the live-attenuated *Mycobacterium bovis* Bacillus Calmette-Guerin (BCG), whose use as a vaccine has constituted an important measure to prevent childhood tuberculosis, but less efficiently confers protection against pulmonary tuberculosis in adults [8]. Moreover, BCG has been shown to impart a variable protective efficacy, possibly due to the application of different BCG sub-strains and vaccination policies across the world [9,10].

In animals, BCG vaccination to prevent tuberculosis has been studied frequently, although it has never been implemented as part of control strategies in livestock. As in humans, BCG vaccination has also resulted in variable protective efficacies at population and individual animal levels. Nevertheless, BCG remains the main candidate to vaccinate cattle and other livestock, due to its affordability and safety record [6]. However, BCG vaccination compromises the specificity of diagnostic tests based on PPD tuberculin, such as the tuberculin skin test, the mainstay of ante-mortem surveillance testing as part of the so-called test and slaughter control strategies. This has been a main reason for BCG vaccination not being implemented for cattle [11,12]. Therefore, a diagnostic test that Differentiates Infected from Vaccinated Animals (DIVA) should also be implemented [13], and only then can the assay or field trial be designed to determine the efficacy of vaccination.

In Chile, a National Plan for the Control and Eradication of Bovine Tuberculosis has been implemented, due to the economic losses that the disease represents for the livestock industry [14]. Similar to other foreign experiences, this plan is based on field diagnoses through the skin tuberculin test and post-mortem examination, including bacteriological isolation and real time PCR as confirmatory tests [14]. The plan works under two different epidemiological zones: the eradication zones, corresponding to low prevalence areas (less than 1% of reactor cattle) in the north and south of the country, and the control zone, which is characterized by a high bTB prevalence (more than 5% of reactor cattle) in the central zone of Chile, between the Coquimbo and Bío-Bío regions [15]. The Metropolitan region, in which the capital city Santiago is located, belongs to this control area, showing a high risk for bTB [15]. Furthermore, the test-and-slaughter strategy in this region has not gained significant adherence in a context of lack of compensations, high bTB prevalence and the consequent reluctance of farmers for culling reactor animals [16]. As expected, the national plan has shown contrasting success in the control zone, encouraging the implementation of a research initiative to determine the protective efficacy of BCG vaccination in dairy farms from the Metropolitan region.

The aim of this work is to describe the efficacy of BCG vaccination in neonatal calves from seven dairy herds located in the metropolitan region of Chile, through a prospective, double-blind, cohort study developed under a natural transmission setting.

## 2. Materials and Methods

### 2.1. Herds

Seven dairy herds were enrolled for the study (H1 to H7), all of them with a high bTB prevalence (76%, 55%, 60%, 56%, 15%, 30% and 44%, respectively). Such prevalence records were available in the sanitary web system of Agricultural and Livestock Service of Chile (SAG), which inputs correspond to collected results from single caudal fold tests (CFT) performed in 2016 by authorized veterinarians, using approved bovine Purified Protein Derivatives (PPDB) antigen (Pronabive^®^, México City, Mexico). A high bTB prevalence was the unique criterion for the inclusion of farms in this study, with all of them located in rural areas from the metropolitan region.

The interferon gamma release assay (IGRA) using DIVA antigens (ESAT-6, CFP-10 and Rv3615c) was authorized as the official test for replacement of the tuberculin skin test on these farms. Reactor cattle remained in herds and were not segregated. Because of this, post-mortem examinations were not performed, and the infection condition was assumed from the DIVA results, interpreting DIVA-positives and DIVA-negatives as reactors (probably infected) and non-reactors (negative), respectively. After farmers were willing to participate and signed the informed consent, the fieldwork started in April 2017.

### 2.2. Animals

The research team scheduled monthly visits to herds, and the farm workers were responsible for the mobilization of animals into pens, where vaccination or sampling was performed.

All Holstein female calves up to 40 days old were recruited for the study, and the randomization by individuals determined that a similar proportion of BCG-vaccinated and control animals was intended in every fieldwork. Animals were always managed under routine conditions of farms. Those animals that died between the inoculation time and 6 months of age were recorded for the calculation of mortality rates in calves.

### 2.3. BCG Vaccination Schedule

Vaccinations were performed between April 2017 and September 2018, totalling 1089 enrolled calves. One group of animals (*n* = 575) was vaccinated subcutaneously in the neck with 2–8 × 10^5^ colony-forming units (CFU) (0.1 mL) of BCG Russia strain (Serum Institute of India, Pune, India), and the other group (*n* = 514) received 0.1 mL of sterile saline (NaCl 0.9%), corresponding to the vaccine diluent. Inoculations were performed alternating animals for vaccinated and placebo groups by a subcutaneous injection in the left side of the neck.

### 2.4. Blood Sampling and IFNγ Release Assay (IGRA)

Animals were sampled at 6, 12 and 18 months post-inoculation. Once 5 mL of peripheral blood was collected in heparinized tubes (BD Vacutainer^®^, Franklin Lakes, NJ, USA), the samples were labelled and maintained at environmental temperature until arriving at the laboratory. IGRA was performed as described previously [16], with blood cultures initiated on the same day of blood sampling. The blood was stimulated with ESAT-6/CFP-10 and Rv3615c peptide cocktails, separately (5 µg/each peptide/mL)(DIVA antigens), avian and bovine PPD (PPDA and PPDB, 250 and 300 IU/mL, respectively), or Pokeweed mitogen (6 µg/mL) (Applied Biosystems^®^Bovigam^®^, Tullamarine, VIC, Australia). PBS was used as control solution. For each antigen, duplicate wells were used (final volume 275 µL per well of 96-well microtiter plates).

After an incubation period at 37 °C for 18 h, plasma supernatants were harvested and stored at −20 °C. Then, plasma samples were thawed, and IFNγ was detected using the Bovigam 2G^®^ Test Kit for cattle (Prionics AG, Tullamarine, VIC, Australia), according to manufacturer recommendations. The cut-off value for the definition of a positive result for DIVA antigens was an optical density at 450 nm difference ≥0.1 after the PBS control values were subtracted from either DIVA cocktail (∆OD_450_). For interpreting results with PPD, an animal was a reactor when the difference between PPDB and PPDA was ≥0.05, after the PBS control values were subtracted from either PPD, in agreement with the Chilean regulations.

### 2.5. Analysis of Results

The efficacy of the vaccine (EV) was calculated using the formula EV(%) = ([Rn − Rv]/Rn) × 100, where Rn and Rv are the rate of incidence in the unvaccinated and vaccinated groups, respectively [17].

The proportion of dead animals in the first 6 months post-vaccination, and the infected animals along the study, were compared between groups using the Fisher’s exact test. The dynamic of the DIVA test negativity along the study was determined in a Kaplan–Meier survival analysis.

The proportion of new *M. bovis*-infected animals was evaluated with the incidence rate, which accounts for the time at risk of infection for each animal of the study. Then, the new infections detected in sampling times were divided by the animal-month at risk in that period. In our conditions, 6 animal-months at risk were assigned to each animal per sampling activity. When infection was detected, 3 animal-months were registered for that period [18].

The IGRA results (∆OD_450_) were compared between vaccinated and control groups with the non-parametric Mann–Whitney test, and between sampling times within groups with the Friedman test. The correlation between EV% values with mortality rates of calves was assessed with the Spearman correlation test (rs). In order to determine correlations between BCG efficacy and the intensity of infection with tuberculous and non-tuberculous mycobacteria (NTM) within herds, the EV% values also correlated with the incidence of PPDB and PPDA reactors in the unvaccinated group of animals.

Incidence rate comparisons between groups and the Spearman correlation test were determined using R version 3.6.1 [19] with “fmsb” and “rcmdr” packages, respectively. The other statistical analyses were carried out using the software Infostat^®^ [20].

## 3. Results

The records of calf mortality between vaccination and 6 months did not show statistical differences between vaccinated (12.9%) and control (14.2%) groups (*p* > 0.05), although high variability was observed among herds (Table 1). Because of these losses, 942 available for sampling animals were included in the analysis of incidence and vaccine efficacy, with 501 and 441 calves in the vaccinated and control groups, respectively.

The overall infection status was determined using the DIVA IGRA, identifying a higher percentage of reactor animals in the control group (182/441, 41.3%) compared to the vaccinated (171/501, 34.1%) group (Table 1, *p* = 0.0261). For these analyses, the DIVA IGRA-positive animals were excluded from subsequent sampling activities and were counted only at the time point of detection. Due to this and other causes of on-farm losses, the total number of animals under the study decreased over time.

**Figure 1:**
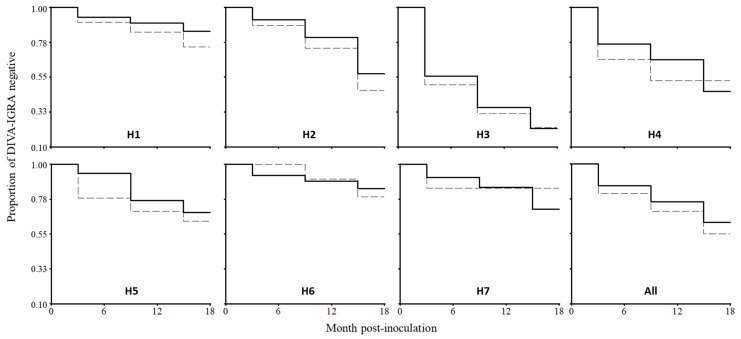
Kaplan–Meier analysis on the comparison of IFN-γ release assay (IGRA) results using DIVA (CFP-10/ESAT-6 and Rv3615c) antigens between BCG-vaccinated (solid lines) and control groups (segmented lines). Tags indicate the herd ID (H1 to H7), and the last graph shows the results obtained with all the animals in the study (All).

The overall incidence rates of bTB were 2.5 (95% CI 2.18 to 2.95) and 3.3 (95% CI 2.83 to 3.79) animals per 100 animal-months at risk in the BCG-vaccinated and control groups, respectively, a difference that was significant (*p* = 0.026) (Table 2). Incidence rates were variable between herds (Table 1), depicting different survival curves in the Kaplan–Meier analysis (Figure 1). The overall calculated vaccine efficacy (EV%) was 22.4% (95% CI 4.0 to 36.4) (Table 2), ranging between 0% and 42.2% between herds (Table 1). The EV% also varied along the sampling times, within and between herds (Table S1), determining the highest overall BCG efficacy at 6 months (27.6%), decreasing at 12 (18.8%) and then maintained at 18 months (18.7%) post-vaccination (Table 2).

The EV% values observed in each herd were negatively correlated with calf mortality rates (rs = −0.71, *p* = 0.04). Contrasting correlations were calculated between EV% and PPDB (rs = −0.22) and PPDA (rs = 0.54) reactors in the unvaccinated group of animals, although without statistical significance (*p* > 0.05).

**Figure 2:**
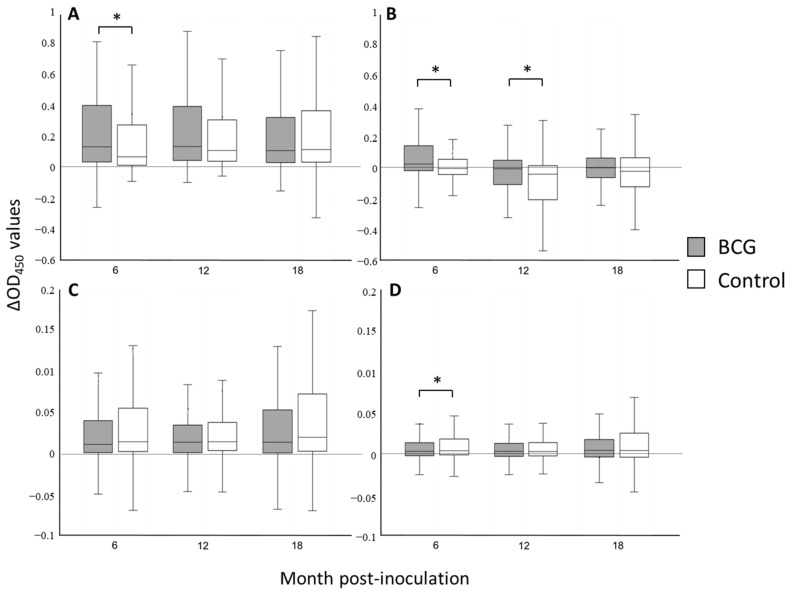
Box-plot diagrams showing IFN-gamma responses at 6, 12 and 18 months post-inoculation in BCG and control groups. The figure shows the IFN-gamma release assay (IGRA) results (∆OD_450_) obtained with (**A**) PPDB minus saline, (**B**) PPD B-A (bovis minus avium), (**C**) CFP-10/ESAT-6 peptide cocktail minus saline and (**D**) RV3615c cocktail minus saline. Within box-plot diagrams, the median is represented with a line, the interquartile range with a box, and the minimum and maximum of the data with the whiskers. (* *p* < 0.05).

The IGRA results with PPDs and DIVA antigens are shown in Table S2 and are depicted in Figure 2. The response after the stimuli with the PPDB tuberculin was significantly higher in the vaccinated group at 6 months (median 0.14, *p* < 0.0001). However, the control group of animals also showed high IFN-γ response at 6 months that was increasing at 12 and at 18 months (*p* < 0.0001) (median values 0.08, 0.12 and 0.13, respectively) (Figure 2A).

The difference between PPD antigens (PPD B-A) was greater in the vaccinated than the control group at 6 and 12 months post-vaccination (*p* < 0.001) (Figure 2B). Furthermore, a significant sensitization with environmental mycobacteria between these months occurred in both groups of animals (Table S3), with a consequent fluctuation in IFN-γ values of the PPD B-A relationship along the study (*p* < 0.0001) (Figure 2B). The incidence of such sensitization with NTM was higher in the control group (*p* = 0.0027, Figure S1), showing a significant increase in the number of PPDA reactors (PPD A-B ≥ 0.05) at 12 months post-vaccination (*p* = 0.0033) (Table S3).

Within IGRA results were observed with DIVA antigens, the median ∆OD_450_ values of the response to ESAT-6/CFP-10 ranged between 0.01 and 0.02 (Figure 2C) and to RV3615c ranged between 0.003 and 0.004 (Figure 2D). In addition, statistical differences were observed between groups of animals with this RV3615c at 6 months post-vaccination (*p* = 0.01) (Figure 2D).

## 4. Discussion

The analysis of *M. bovis* BCG effects in cattle as a complementary tool for the prevention of bTB has been undertaken for several decades in a myriad of studies. Recently, a meta-analysis addressing the bibliographic background, including 24 articles published between 1972 and 2018, suggests an overall vaccine efficacy of 25% to avoid lesions and/or the isolation of *M. bovis* [5], although a wide range in BCG efficacy (0 to 75%) has been reported [21]. A diversity of experimental and natural settings, doses and BCG strains, ages, breeds and routes of administration were included in the meta-analysis, making difficult the comparisons between studies and, consequently, the identification of optimal conditions for vaccination in cattle [5].

With more similitudes to this work, field trials in Ethiopia and Mexico have been performed to determine BCG vaccine efficacy in young calves, applying a subcutaneous inoculation of 1 × 10^6^ colony-forming units (CFU) of BCG. In these experiences, a protective efficacy ranging from 23% to prevent visible lesions and 68% to prevent carcass condemnation was calculated when animals were followed for 12 months after vaccination [22,23,24,25]. Compared with these reports, our experience is in the low range of protection, with 22.4% of BCG efficacy in animals followed by 18 months, and 24.4% when the follow-up is for 12 months post-vaccination (data not shown), although being within the protection interval was determined by the meta-analysis of Srinivasan et al. [5].

Unfortunately, a limitation of this work is that the evaluation of infection status was only based on detecting the specific cellular immune responses against infection through the DIVA-IGRA test, since herds were enrolled with the assumption that the animals would be maintained, managed and culled under own-farm routines and decisions. Although sensibility limitations would exist and the infection can be underestimated, comparative genome and transcriptome analysis have determined that the peptides belonging to CFP-10, ESAT-6 and RV3615c antigens, when used simultaneously, attain a similar or even better performance than the traditional tuberculin test [26,27,28,29]. Due to the misclassification of positive animals occurring in both vaccinated and non-vaccinated groups of animals, the observed differences in incidence rates can be effectively attributed to vaccination. Furthermore, our results show that the direct full protection conferred by the BCG vaccine, due to those partially protected animals (infected with a reduced pathology), are detected by DIVA-IGRA.

What determines the variation in the vaccine protection between different reported field trials could be related to several environmental-, host-, pathogen- and vaccine-related factors. This work, which was held on seven independent herds from the metropolitan region of Chile, also detected a range of protective efficacies achieved by the BCG vaccine (Table 1), despite using the same vaccine strain, inoculation route, dose, age and breed of animals. What we could observe as possible factors associated with such contrasting results between herds, were the mortality rates of calves, and the incidences of DIVA and PPDA reactors.

While the proportion of dead calves was high in all studied farms (Table 1), it is remarkable that better EV% were obtained in those herds with the lowest levels of calf mortality. The significant and negative correlation between these variables (rs = −0.71, *p* = 0.04) suggests that the general health status of the animals around the vaccination period, and consequently the integrity of their immune system when exposed to BCG antigens, is essential for achieving vaccine-associated protection. A high proportion of neonatal calf diarrhoea and respiratory disease was recorded by producers in these mortality events, although no etiological diagnosis was made. Therefore, any effort to prevent bTB and other diseases through vaccination will depend on farm management for preserving the sanitary condition of young animals.

A second factor under analysis was the intensity of infection to which the vaccinated animals were exposed, mainly represented by seeder-infected animals from the unvaccinated (control) group. This factor has been suggested as a source of variability in protection, which correlates negatively with vaccine efficacy [22,24]. In line with this background, our results showed that the correlation between EV% and the incidence of bTB reactors in the unvaccinated group was negative (rs = −0.22, *p* > 0.05), although of less magnitude than the analysis of calf mortality. However, other studies were performed with similar or even higher pressures of infection, obtaining better BCG EV% results [25,30], suggesting that this factor should be considered in the equation, although apparently with less relevance to explain observed differences between herds or field trials.

The other variable is the intensity of infection with *M. avium* or environmental mycobacteria, which has been associated with reductions in BCG efficacy due to immune interference in vaccinated animals [31,32]. In this work, the incidence of NTM, which is assumed from PPDA reactors in the control unvaccinated group (Table S3), was positively correlated with EV% results (rs = 0.54, *p* > 0.05), making such deleterious effects in the vaccine-associated immunity improbable and, on the contrary, suggesting some contribution for protection against *M. bovis* infection, as has been reported elsewhere in cattle [33]. Furthermore, it seems that the BCG vaccination also confers protection against NTM exposition, since we observed a higher incidence of PPDA reactors (PPD A-B ≥ 0.05) in the unvaccinated group of animals (Figure S1, *p* = 0.0027), especially at 12 months post-vaccination (Table S3). The group of NTM includes a wide diversity of opportunistic pathogens that can grow and persist in almost every environmental component [34], and the relatedness of the species circulating in dairy farms to virulent *M. bovis* or BCG will probably influence vaccine efficacy in these animal populations [5]. 

A finding that attracts more agreement among the studies is that protective immunity does not last for the lifetime of the animals, waning between 12- and 24-months post-inoculation [6,35]. In this work, we observed such a decline in the EV% between 6- and 12-months post-vaccination (Table 2). Again, such a finding was variable between herds, because some of them presented a declined (H2, H3, H5, H7) or increased (H1, H6) efficacy during the sampling times of this study (Table S1), with different dynamics in the incidence and DIVA test negativity along the study (Figure 1), without an evident causal factor. Stress from intensive or deficient management might accelerate the decrease in BCG-induced immunity and promote intra-herd transmission of *M. bovis* in livestock [36]. Thus, the experimental evidence that suggests revaccination after two years [31,37] seems advisable under certain field conditions, and should be adjusted according to the local herd bTB incidence. However, this two-dose strategy remains to be evaluated under field conditions in dairy cattle.

The cell-mediated immune response was evaluated using the IGRA, with PPDs and DIVA antigens, which was performed from 6 month of age, as recommended to avoid the nonspecific results associated with the IFN-γ secretion of NK cells in younger animals [38]. As expected, the IFN-γ response to the PPDB antigen was higher in the vaccinated group, a difference that was observed as significant at 6 months (*p* < 0.0001) (PPDB-PBS, Figure 2A), in agreement with earlier studies using IGRA or skin tests [24,37]. Hence, the interference of BCG with the diagnosis based on PPD tuberculin lasted between 6 and 12 months, supporting the need of alternative antigens to be included as DIVA reagents, at least during the first year post-vaccination [26].

The median of IFN-γ response to PPDB was increasing in the control group alongside the study (*p* < 0.0001) (Figure 2A), despite a different dynamic in the bTB incidence measured with DIVA antigens (Table 2). This contrast may result from the exposition to NTM, which generates a cross-reactivity with the response to PPDB [39]. The resulting lower specificity of the bTB diagnosis using this antigen may be significant in field conditions, with a high occurrence of environmental mycobacteria, such as the observed in this study (Table S3).

The difference between PPD antigens (PPD B-A) was higher in the vaccinated group of animals at 6 months, reflecting the mentioned BCG effect on the IFN-γ response to PPDB (Figure 2B). However, at 12 months, the PPD B-A relationship was also different between groups (*p* < 0.05), as a result of a significant exposition to environmental mycobacteria that occurred between 6 and 12 months. The difference emerged because this exposition sensitized the control group more significantly (*p* < 0.05), due to a protective effect conferred by BCG in vaccinated animals (Figure S1), as has been previously observed in children [40]. Although such cross-protection requires further studies to characterize changes in pathology or diseases associated with these NTM, this finding suggests a secondary beneficial effect of BCG vaccination in cattle.

The IFN-γ response induced by DIVA antigens showed independence from the vaccination condition or from the *M. avium* incidence (Figure 2C,D), demonstrating specificity under these epidemiological conditions. The ESAT-6/CFP-10 peptide cocktail elicited higher IFN-γ median ∆OD_450_ values than RV3615c in reactor animals, although this antigen allowed for the detection of differences in IGRA between vaccinated and control groups at 6 months (*p* = 0.01), when the incidences of both cohorts also showed a significant difference (*p* = 0.047, Table 2). Hence, these three antigens must be included in a DIVA test when a BCG vaccination campaign is implemented, or when environmental mycobacteria could influence the test accuracy using traditional PPD antigens.

## 5. Conclusions

In conclusion, the direct vaccine efficacy conferred by the BCG vaccine in dairy calves belonging to seven herds in central Chile was low but significant when analysed through IGRA results, supporting the use of BCG to contribute in bTB control efforts in this high prevalence area. Elucidating risk factors that were associated with variable vaccine efficacies between herds, and determining post-mortem evidence of protection remains as relevant data to be determined in future studies. Furthermore, dairy farmers must urgently address the high mortality of calves observed in studied herds as a critical condition to attain the protective immune response conferred by BCG, which may be implemented as a complementary tool in the control and eradication of bTB in high prevalence areas.

## Figures and Tables

**Table 1:** Calf mortality, reactor animals, incidence rates (IR%) and efficacy of vaccine (EV%) in BCG-vaccinated and control animals per dairy herd.

Herd ID	Vaccination Status	N° Dead Calves (%) *	N° Positives/Total (% Positives)			IR%	EV%
			6 M	12 M	18 M		
H1	BCG	11 (6.5)	10/159 (6.3)	6/147 (4.1)	7/120 (5.8)	0.9	42.2
	Control	14 (8.8)	14/146 (9.6)	9/128 (7.0)	11/97 (11.3)	1.5	
H2	BCG	15 (9.7)	11/140 (7.9)	16/126 (12.7)	28/96 (29.2)	2.7	24.5
	Control	15 (10.3)	15/130 (11.5)	19/115 (16.5)	30/81 (37)	3.6	
H3	BCG	10 (11.2)	35/79 (44.3)	15/41 (36.6)	8/21 (38.1)	8.6	8.5
	Control	11 (12.6)	38/76 (50)	13/35 (37.1)	5/16 (31.3)	9.4	
H4	BCG	15 (28.3)	9/38 (23.7)	3/22 (13.6)	5/16 (31.3)	4.1	7.8
	Control	15 (33.3)	10/30 (33.3)	4/19 (21.1)	0/10 (0)	4.5	
H5	BCG	0 (0)	2/34 (5.9)	6/32 (18.8)	2/20 (10)	2.1	25.9
	Control	2 (8)	5/23 (21.7)	2/18 (11.1)	1/11 (9.1)	2.8	
H6	BCG	14 (33.3)	2/28 (7.1)	1/24 (4.2)	1/20 (5)	1.0	17.1
	Control	10 (30.3)	0/23 (0)	2/21 (9.5)	2/16 (12.5)	1.2	
H7	BCG	9 (28.1)	2/23 (8.7)	1/15 (6.7)	1/6 (16.7)	1.6	0
	Control	6 (31.6)	2/13 (15.4)	0/7 (0)	0/2 (0)	1.6	

* Dead animals between vaccination and 6 months post-vaccination time points.

**Table 2:** Incidence rates and efficacy of the vaccine (EV%) at 6, 12 and 18 months post-inoculation.

Month PI	Incidence Rates (%)			EV%
	BCG	Control	*p* Value	
6 M	2.5	3.5	0.047	27.6
12 M	2.1	2.6	0.371	18.8
18 M	3.2	3.9	0.311	18.7
Total	2.5	3.3	0.026	22.4

## Data Availability

The data that support the findings of this study are available in the Supplementary Materials of this article.

## Supplementary Materials

The following supporting information can be downloaded at: https://www.mdpi.com/article/10.3390/ani12091083/s1, Figure S1: Kaplan–Meier analysis on the comparison of environmental mycobacteria incidence between BCG-vaccinated and control groups. Table S1: Efficacy of the BCG vaccine (EV%) at 6, 12 and 18 months post-vaccination in each dairy herd. Table S2: Interferon gamma release assay (IGRA) results at 6, 12 and 18 months post-inoculation, using PPDB, PPDA, ESAT-6/CFP-10 and Rv3615c antigens. The OD_450_ nm values of the PBS controls were already subtracted from the OD_450_ nm values of antigens. Table S3: Reactor animals and incidence rates (IR%) of *Mycobacterium avium* (∆OD_450_ PPDA-PPDB ≥ 0.05) in BCG-vaccinated and control animals per dairy herd.
