# Supplementary material for: Vaccination of Calves with the Mycobacterium bovis BCG Strain Induces Protection against Bovine Tuberculosis in Dairy Herds under a Natural Transmission Setting
Source: Animals (Basel). 2022 Apr 22;12(9):1083. doi: 10.3390/ani12091083 (PMC9102018; doi:10.3390/ani12091083)
Supplement: Supplementary file 1 [file animals-12-01083-s001.zip › animals-1613848-supplementary.pdf]

Table S1. Efficacy of the BCG vaccine (EV%) at 6, 12 and 18 months post-vaccination in each dairy herd

Herd ID	EV%		
	6M	12M	18M
H1	34.4	41.4	48.7
H2	31.3	23	21.1
H3	11.4	1.3	0
H4	28.8	35.5	0
H5	72.8	0	0
H6	0	55.8	60
H7	43.5	0	0
Total	27.6	18.8	18.7

Table S3. Reactor animals and incidence rates (IR%) to *Mycobacterium avium* (ΔOD_{450} PPDA-PPDB ≥0.05) in BCG vaccinated and control animals per dairy herd.

Herd ID	Vaccination status	N° positives/total (% positives)			IR%
		6M	12M*	18M	
H1	BCG	30/159 (18.9)	45/120 (37.5)	19/64 (29.7)	5.3
	Control	31/146 (21.2)	66/102 (64.7)	8/24 (33.3)	8.0
H2	BCG	18/140 (12.9)	21/110 (19.1)	9/65 (13.8)	2.8
	Control	22/130 (16.9)	26/93 (28.0)	6/44 (13.6)	3.8
H3	BCG	10/79 (12.7)	16/34 (47.1)	2/7 (28.6)	4.4
	Control	20/76 (26.3)	8/20 (40.0)	3/4 (75.0)	6.1
H4	BCG	7/38 (18.4)	1/17 (5.9)	4/14 (28.6)	3.1
	Control	7/30 (23.3)	6/16 (37.5)	3/5 (60.0)	6.2
H5	BCG	13/34 (38.2)	8/19 (42.1)	2/6 (33.3)	8.1
	Control	10/23 (43.5)	2/19 (22.2)	3/4 (75.0)	8.8
H6	BCG	6/28 (21.4)	5/19 (26.3)	1/12 (8.3)	3.8
	Control	2/23 (8.7)	2/19 (10.5)	2/12 (16.7)	2.0
H7	BCG	2/23 (8.7)	3/13 (23.1)	1/5 (20.0)	2.6
	Control	2/13 (15.4)	0/6 (0)	1/1 (100)	2.7
Total	BCG	86/501 (17.2)	99/332 (29.8)	38/173 (22.0)	4.2
	Control	94/441 (21.3)	110/265 (41.5)	26/94 (27.7)	5.6

*p=0.0033

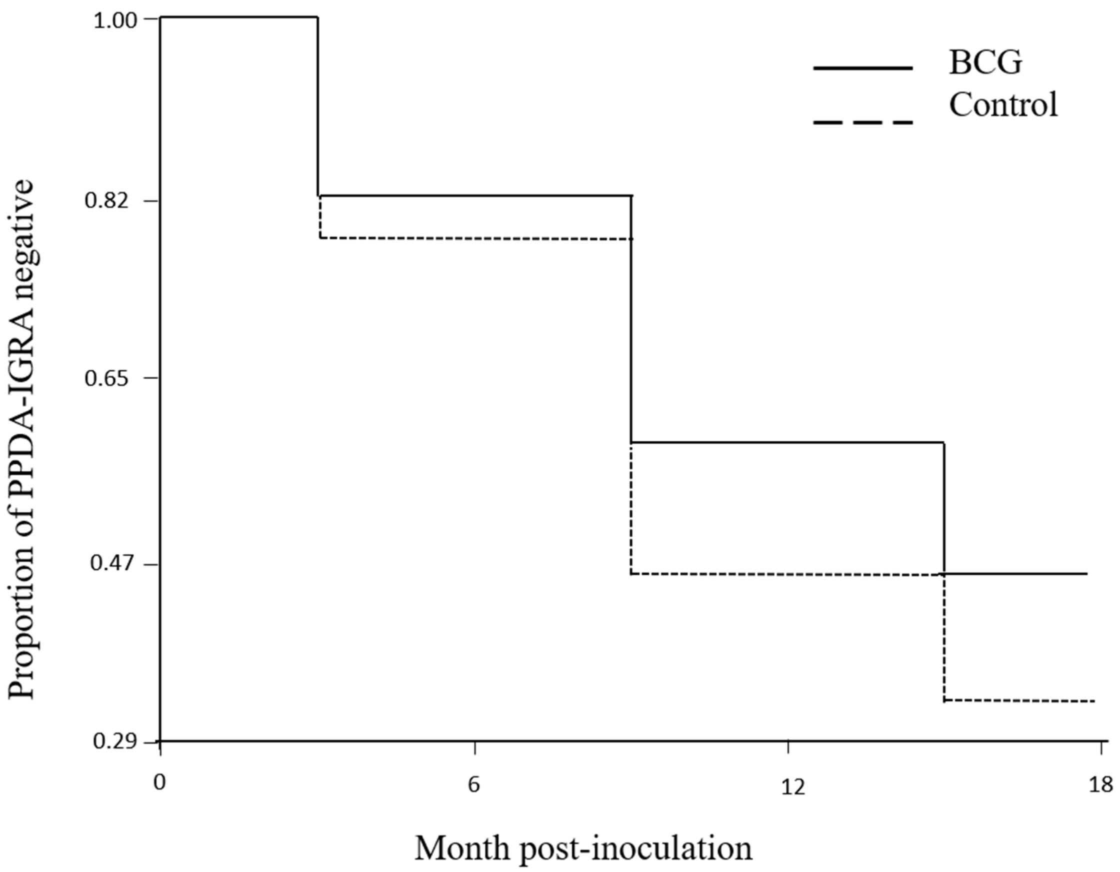

Figure S1. Kaplan-Meier analysis on the comparison of environmental mycobacteria incidence between BCG vaccinated and control groups, determined through the IFN-γ release assay (IGRA) using PPD antigens (PPDA-PPDB ≥0.05 OD_{450} nm). The animals that received the BCG vaccine have a significantly longer *M. avium* infection-free status than the control group (p=0.0027).
